# Abnormal Functional Resting-State Networks in ADHD: Graph Theory and Pattern Recognition Analysis of fMRI Data

**DOI:** 10.1155/2014/380531

**Published:** 2014-08-31

**Authors:** Anderson dos Santos Siqueira, Claudinei Eduardo Biazoli Junior, William Edgar Comfort, Luis Augusto Rohde, João Ricardo Sato

**Affiliations:** ^1^Center of Mathematics, Computation and Cognition, Universidade Federal do ABC, Avenida dos Estados 5001, 09210-580 Santo Andre, SP, Brazil; ^2^NIF-LIM44, Institute of Radiology, Hospital das Clinicas, University of Sao Paulo, Avenida Dr. Enéas de Carvalho Aguiar, 05403-900 Sao Paulo, SP, Brazil; ^3^Department of Psychiatry, Federal University of Rio Grande do Sul, Rua Ramiro Barcelos 2350, 90035-903 Porto Alegre, RS, Brazil; ^4^National Institute of Developmental Psychiatry for Children and Adolescents, Brazil

## Abstract

The framework of graph theory provides useful tools for investigating the neural substrates of neuropsychiatric disorders. Graph description measures may be useful as predictor variables in classification procedures. Here, we consider several centrality measures as predictor features in a classification algorithm to identify nodes of resting-state networks containing predictive information that can discriminate between typical developing children and patients with attention-deficit/hyperactivity disorder (ADHD). The prediction was based on a support vector machines classifier. The analyses were performed in a multisite and publicly available resting-state fMRI dataset of healthy children and ADHD patients: the ADHD-200 database. Network centrality measures contained little predictive information for the discrimination between ADHD patients and healthy subjects. However, the classification between inattentive and combined ADHD subtypes was more promising, achieving accuracies higher than 65% (balance between sensitivity and specificity) in some sites. Finally, brain regions were ranked according to the amount of discriminant information and the most relevant were mapped. As hypothesized, we found that brain regions in motor, frontoparietal, and default mode networks contained the most predictive information. We concluded that the functional connectivity estimations are strongly dependent on the sample characteristics. Thus different acquisition protocols and clinical heterogeneity decrease the predictive values of the graph descriptors.

## 1. Introduction

Attention-deficit/hyperactive disorder (ADHD) is a neurodevelopmental disorder with a prevalence of around 5.3% in children and adolescents [1]. It is characterized by cognitive and behavioral impairments associated with inattention and/or hyperactivity and impulsivity symptoms [2]. The most frequent and investigated ADHD phenotypes are the ones with predominance of inattentive symptoms and a group that combines inattention and hyperactivity/impulsivity. As for most mental disorders, the etiological bases and neural substrates of ADHD are far from being fully understood. The search for structural or functional neural correlates of ADHD, and consequently for potential biomarkers of the disorder, is crucial in the pursuit of its prevention, early detection and more effective treatment [3, 4]. For this purpose, the combination of machine-learning techniques for pattern recognition and resting-state functional neuroimaging data is a particularly promising approach [5].

Graph theoretical analysis is an emerging component in the field of connectomics and brain network analysis based on neuroimaging data [6, 7]. Descriptors derived from graph theory are measurements quantifying different characteristics of the network organization. When applied to resting-state fMRI data, graph theoretical measures may be used to enhance the understanding of resting-state network (RSN) dynamics [8]. RSNs are characterized by consistent correlations with the spontaneous fluctuations of the BOLD signal among certain brain regions. Among the diffuse RSNs identified via fMRI analysis, specifically sensory-motor, frontoparietal, basal ganglia, and default mode networks have been implicated in ADHD pathophysiology [9]. Currently, abnormal interactions within distinct RSNs have been identified as a key factor in contributing to various neuropsychiatric disorders [10], in particular within the default mode network (DMN) [11, 12].

Pattern recognition methods based on machine learning techniques have shown to be a promising approach to the analysis of neuroimaging data [13]. Support vector machines (SVMs) [14] are one of the most frequently used methods in this field, given their robust properties when dealing with high dimensional multivariate data in addition to providing predictions for each individual case. In other words, given a set of features (e.g., brain measurements) and a label (e.g., healthy and patient), SVMs are used to learn a function, which maps the set of features to their respective labels within a training dataset. Thus, given a new set of features produced from an unseen observation, SVMs are able to provide a predicted label for this novel observation.

Graph theory descriptors can be used as predictor variables (i.e., features) in a machine-learning framework. Merging graph theoretical approaches and machine learning techniques might provide a better-adjusted way to scrutinize the impairment of RSNs in ADHD as well as mapping predictions to a single individual case. In this study, we investigated the use of network centrality measures as predictive features to discriminate between typical developing children and ADHD patients with both inattentive and combined presentations. In addition, we investigated possible differences between inattentive and combined ADHD groups. The ADHD-200 dataset [15] formed the basis of our analysis. We aimed at evaluating three issues: (i) the mean classification score ([sensitivity + specificity]/2) across distinct acquisition sites; (ii) the classification score site-by-site (i.e., only the data within each site are used to train and test the classifier) with a global classification (i.e., using the data of all sites in a joint analysis); (iii) brain regions (i.e., network nodes) containing the greater amount of predictive information to discriminate between the groups. We hypothesize that frontoparietal, sensory-motor, and default mode network nodes will have a more relevant predictive value in the classification. This hypothesis relies on the potential association between abnormalities in resting-state networks and the main symptoms of ADHD.

## 2. Materials and Methods

### 2.1. Data and Image Preprocessing

The publicly available resting-state fMRI data from the ADHD-200 Consortium were used in the present study. The images were acquired at five different sites: Peking University, Kennedy Krieger Institute, NeuroIMAGE sample, New York University Child Study Center, and Oregon Health & Science University (OHSU). The subject sample consisted of 609 subjects, 340 controls (mean age [standard deviation] − 11.59 [2.86] years; 180 males), and 269 patients with ADHD according to DSM-IV-TR criteria (mean age [s.d.] − 11.58 [2.88] years; 215 males). Among the total number of ADHD patients, 159 fulfilled the criteria for the inattentive type (mean age [s.d] − 11.24 [3.05] years, 130 males), while 110 were classified as the combined type (mean age [s.d.] − 12.08 [2.55] years, 85 males).

All research protocols from institutes contributing to the ADHD-200 Consortium received local approval by their respective IRB. All the data distributed via the International Neuroimaging Data-sharing Initiative (INDI) are fully anonymized in accordance with HIPAA Privacy Rules. Further details concerning the sample and scanning parameters can be obtained by request to the ADHD-200 Consortium.

 Step-wise data preprocessing was previously conducted by the NeuroBureau community using the Athena pipeline and consisted in the systematic and homogeneous processing of all resting-state fMRI data. The following steps were carried out: exclusion of the first four EPI volumes; slice time correction; deobliquity of the dataset; head motion correction using the first volume as a reference; exclusion of voxels at non-brain regions by masking the volumes; averaging the EPI volumes to obtain a mean functional image; coregistration of this mean functional image to the subjects' correspondent anatomical image; spatial transformation of functional data into template space; extraction of BOLD time series from white matter and cerebrospinal fluid using masks obtained from segmenting the structural data; removing trend and motion effects through linear multiple regression; temporal band-pass filtering; spatial smoothing using a Gaussian filter.All preprocessed images are available at the website http://neurobureau.projects.nitrc.org.

### 2.2. Connectivity Analysis and Graphs

A representative set of 400 brain-wide regions of interest (ROIs) was chosen for defining the network nodes used for connectivity analysis and the construction of the graphs. The ROIs were determined by using the method developed by Craddock et al.  [16] based on the fMRI data of 650 subjects. This atlas is publicly available at http://www.nitrc.org/plugins/mwiki/index.php/neurobureau:AthenaPipeline. The Pearson correlation coefficient between each pair of ROIs was calculated and regarded as a proxy of functional connectivity. The correlation matrix was equated with the adjacency matrix of an undirected and weighted graph. Meanwhile, binary adjacency matrices were built for each subject by applying three different cut-off values (0.1, 0.15 and 0.25) to the correlation matrix. The cut-offs were defined within this particular range since the network becomes too fragmented and granular to allow a proper graph analysis for higher cut-off values [17]. We evaluated the predictive power from both weighted and unweighted graphs. The following centrality measures of the nodes in the weighted graph were calculated: degree, closeness [18], betweenness [19], eigenvector, and Burt's constraint [20]. The degree, closeness, and betweenness were also calculated for the unweighted graphs.

The mathematical definitions of these measures are described in Table 1 where *N* is the set of all nodes and edges within a network and *n* is the number of nodes. An edge between two nodes *i* and *j* is represented by *a*_*i*,*j*_. In the undirected graph case, *a*_*ij*_ = 1 if there is a connection between the nodes *i* and *j*; otherwise, *a*_*ij*_ = 0. In betweenness definition, *ρ*_*hj*_ is the number of shortest paths between *h* and *j*, and *ρ*_*hj*_(*i*) is the number of shortest paths between *h* and *j* passing through *i*. In eigenvector definition, *l* is a constant. Note that eigenvector and Burt's constraint are definable only for weighted graphs.

Degree is a straight and intuitive way to quantify nodes centrality, and it is defined as the number of edges connected to a particular node. The closeness centrality is the average distance between a given node and all other nodes of the network. Betweenness quantifies the influence of a node and is defined as the number of shortest paths passing through it. The basic rationale underlying eigenvector centrality is that connections with more central nodes increase the nodes influence in the network. Hence, different weights are attributed to a vertex depending on the centrality of the connected nodes. Finally, Burt's constraint value is inversely proportional to the number of connections of a node and increases with the number of strong mutual connections [20]. The uses and interpretations of graph theoretical measures in the context of fMRI studies were the central topic in an excellent previous review [7]. All analyses were performed in the* R* platform for Computational Statistics (*R* Project for Statistical Computing) (http://www.r-project.org/) using the* R igraph* package.

### 2.3. Classifier Implementation and Identification of Discriminative ROIs

The centrality measures of each graph's nodes were used as features (i.e., predictor variables) in an independent classification analysis. Classification was performed using a linear support vector machine (SVM) algorithm [14]. The rationale behind SVM is that the determination of the boundary defined by the predictor variables should maximize the separation margin between the two groups to be classified. Accuracy of the classification model was estimated via a leave-one-subject out cross-validation procedure. The classifications were based on the discrimination between typical developing children compared to ADHD patients (both inattentive and combined, and a comparison between the ADHD-inattentive and ADHD-combined types. For each graph descriptor, two distinct analyses were carried out: (i) an independent site-by-site classification using the data within a single site to train and test the SVM (leave-one-subject-out score) and (ii) a joint analysis concatenating the data strings from all sites into a single classification.

Finally, in order to identify the most discriminative regions, we built brain maps highlighting the 5% brain regions with greater predictive values. We used the approach proposed by Mourão-Miranda et al. [21] and Sato et al. [22]. In brief, the decision function of the linear SVM used to predict the group of each subject is a hyperplane equation. This equation is defined by a constant and a set of coefficients, each one associated to an input feature (i.e., a brain region defined by the ROIs). During the classifier training, these parameters are tuned in order to define the optimum hyperplane for separating the data. We then used the absolute values of these hyperplane coefficients (taking into account the training with all subjects and not the leave-one-out procedure) to rank the features and highlight the top 5% most discriminative brain regions.

## 3. Results

### 3.1. Classifier Accuracy

Table 2 depicts the scores for the between-group condition comparing typical developing children with ADHD patients. The highest score obtained via site-by-site analysis was 73% using weighted betweenness at the OHSU site. However, this finding was not replicated at the other sites. In the whole-sample analysis the highest score was 58%, achieved with eigenvector centrality.

Table 3 shows the scores for the discrimination analysis between inattentive and combined ADHD subtypes. This analysis was more promising and several measures achieved scores greater than 65% across multiple sites. The highest score obtained via site-by-site analysis was 77% when using the degree measure with unweighted graphs (with a 0.15 cut-off) at OHSU. The highest score in whole-sample analysis was 61%, achieved when using unweighted degree (with a 0.25 cut-off).

**Figure 1:**
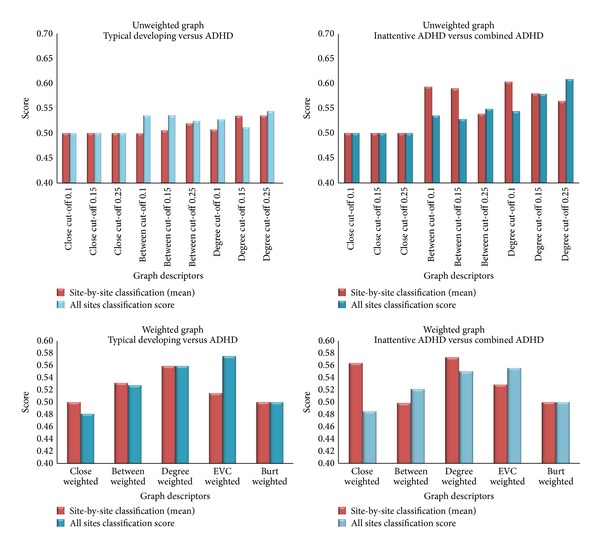
Classification scores ([specificity + sensitivity]/2) for each centrality measure.

Interestingly, the mean score (across sites) and the score from whole-sample classification were very similar, except when using betweenness and degree in unweighted graphs (Figure 1). In this exception, the mean score was greater than the whole sample classification score.

### 3.2. Brain Regions with Higher Predictive Value

**Figure 2:**
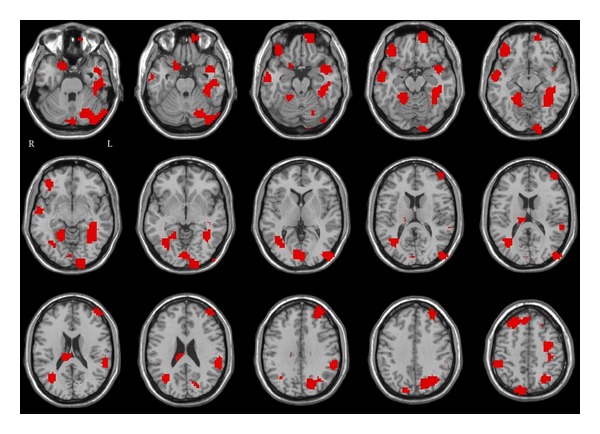
Discriminant regions for betweenness centrality (weighted graph) in typical developing versus ADHD classification at OHSU.

Regarding the identification of the brain regions with greater contribution to prediction, we chose only the classifications with accuracy above 70%.  Figure 2 illustrates the discriminant regions for weighted betweenness centrality in healthy versus ADHD groups at OHSU. Several cerebellar and cortical regions were observed including left cerebellum, cerebellar vermis, bilateral occipital cortex, left inferior temporal gyrus, left parietal cortex, right dorsolateral prefrontal cortex, and left frontal pole.

**Figure 3:**
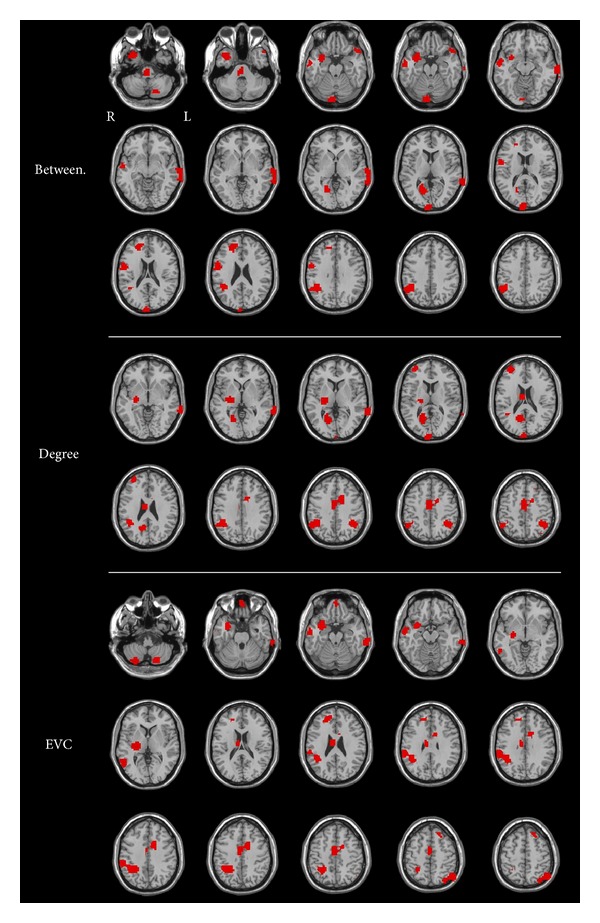
Discriminant regions for unweighted betweenness, weighted degree, and weighted eigenvector centrality in the classification between ADHD types at OHSU.

Figure 3 depicts the regions in which centrality measures contributed to the classification of the ADHD types in the OHSU sample. Betweenness centrality contributed most to classification in the following brain regions: thalamus, left cerebellar cortex, right occipital cortex, right temporal cortex, right precuneus, and right dorsomedial prefrontal and parietal cortices. The brain regions in which degree centrality contributed mostly to classification of ADHD types are also depicted in Figure 3. They include the right temporal and frontal cortices, precuneus and bilateral sensory-motor cortex, dorsal anterior cingulate cortex (dACC), and bilateral parietal regions. In the case of eigenvector centrality, the highest classification scores were obtained in orbitofrontal cortex (OFC), dACC, bilateral temporal cortex, right parietal cortex, motor areas, basal ganglia, and bilateral cerebellum.

## 4. Discussion

At present, resting-state fMRI is a well-established tool for the assessment of spontaneous brain activity. Graph theoretical measures provide a suitable framework for the investigation of the structures of complex neural networks. In addition, the application of machine-learning algorithms has been of great impact on developing more advanced neuroimaging studies of psychiatric disorders [13]. In the present work, we aimed to explore the use of graph-derived measures of resting-state BOLD signal as features to discriminate between ADHD types and healthy subjects. In order to estimate the “real-world” reproducibility of the classification procedure, we analyzed data collected at five distinct sites, which differed in terms of MRI scan specifications and acquisition parameters. Finally, we mapped the brain regions in which centrality graph-derived measures showed the greatest contribution to classification. This mapping could provide some insight into the pathophysiological mechanisms of ADHD from a network analysis perspective.

When the whole sample was used, none of the centrality measures had a relevant predictive power beyond chance. However, significant prediction values were observed at the OHSU site. Thus both within- and between-site variability have a negative impact on the extraction of predictive information and consequently on classification. In the OHSU sample, betweenness centrality measures contained predictive information for the classification of ADHD and control subjects with a score of 73%. After an extensive analysis of sample characteristics and acquisition parameters, we hypothesize that the classification score at OHSU was higher than the other scores for two main reasons: (i) the sample was approximately balanced between typically developing controls (42 subjects) and ADHD patients (35 subjects), while the group sizes were very different at the other sites; (ii) OHSU EPI acquisition has the largest voxel size (3.8 mm) and the 3T system was equipped with a 12 channels head coil (as opposed to 8) which increases the signal-to-noise ratio.

When the 5% nodes with greater predictive values were mapped, a sparse pattern of brain regions was observed. In fact, widespread brain alterations in ADHD are supported by findings of impaired interregional connectivity between the nodes of large-scale functional networks (reviewed in [9]), and both task-related and resting-state fMRI studies described atypical activations in frontal, temporal, and parietal lobes as well as in cerebellum [23–25].

A promising finding was observed for the degree centrality in the whole sample analysis on the classification of the disorder types. In the within-site analyses, relatively high scores were observed for degree, betweenness, and eigenvector centralities. However, as the sample size is smaller in these cases, variability is increased. Moreover, the mean scores of within-site analyses were almost identical to the ones from the whole sample analysis. Brain regions mapped for betweenness measures included nodes of the right frontoparietal network. This network has been implicated in attentional and executive processes and is thought to be impaired in ADHD. Cubillo et al. [23] have shown reduced interregional functional connectivity between frontoparietal network nodes during a stop and switching task in ADHD patients when compared to control subjects. Of particular note is the thalamus, which forms part of this attentional network [26, 27], and consequently may play a key role in ADHD. In fact, reduced regional activations in bilateral thalami have been reported in ADHD. Additionally, reduced connectivity between the thalamus and right prefrontal region, occurring concurrently with increased connectivity between the thalamus and occipital lobes, has been found in ADHD in an fMRI study using a sustained attention task [28]. Interestingly, betweenness is the number of shortest path lengths that pass through a node, which is consistent with the purported structural position of the thalamus as a relay to the whole cortex sheet. We speculate that a high betweenness value for the nodes of the attentional network is compatible with the function of switching attention focus to different stimuli or tasks.

The measure of degree centrality, when applied to the separation between ADHD types, produced the highest classification scores in areas of the sensory-motor network and of the DMN, mainly in parietal cortex and the precuneus. These findings are in agreement with our hypothesis, based on consistent results in the literature [9]. In fact, it is quite intuitive that motor network connectivity should be altered in a disorder characterized by hyperactivity. It is coherent that the measure of degree centrality (the number of nodes that connect to a given node) contains more discriminative information in these areas, since the motor network fundamentally comprises the output of the central nervous system. It is also expected that motor regions contain information which enables discrimination between inattention with or without hyperactivity. The eigenvector centrality was also found to contribute more to classification within the motor network, as well as within orbitofrontal cortex, dorsal anterior cingulate cortex, parietal regions, basal ganglia, and the cerebellum. Orbitofrontal areas have been classically implicated in impulse control mechanisms and appear to have impaired activation in ADHD patients [26]. Finally, alterations of DMN activity have also been proposed as a key part of ADHD pathophysiology [29]. In summary, functional networks implicated in attention, hyperactivity, and impulsivity contained predictive information for the discrimination between ADHD inattentive and combined subtypes.

In conclusion, a novel approach of applying graph theoretical measures was shown to be useful for testing our hypothesis regarding resting-state network impairment in ADHD disorder. In particular, distinct patterns of network dysfunction were evident for both inattentive and combined ADHD subtypes. The classification scores for discriminating between ADHD and healthy subjects were close to chance. Clearly, within-site analysis improves prediction levels when compared to whole sample analysis, suggesting that heterogeneity across the sites may strongly limit the application of the method as a potential clinical support. The functional connectivity estimation is strongly dependent on the samples' characteristics. Thus, in order to advance the pathophysiological knowledge of ADHD, we emphasize the importance of further multicentric studies with more homogeneous acquisitions.

## Figures and Tables

**Table 1:** 

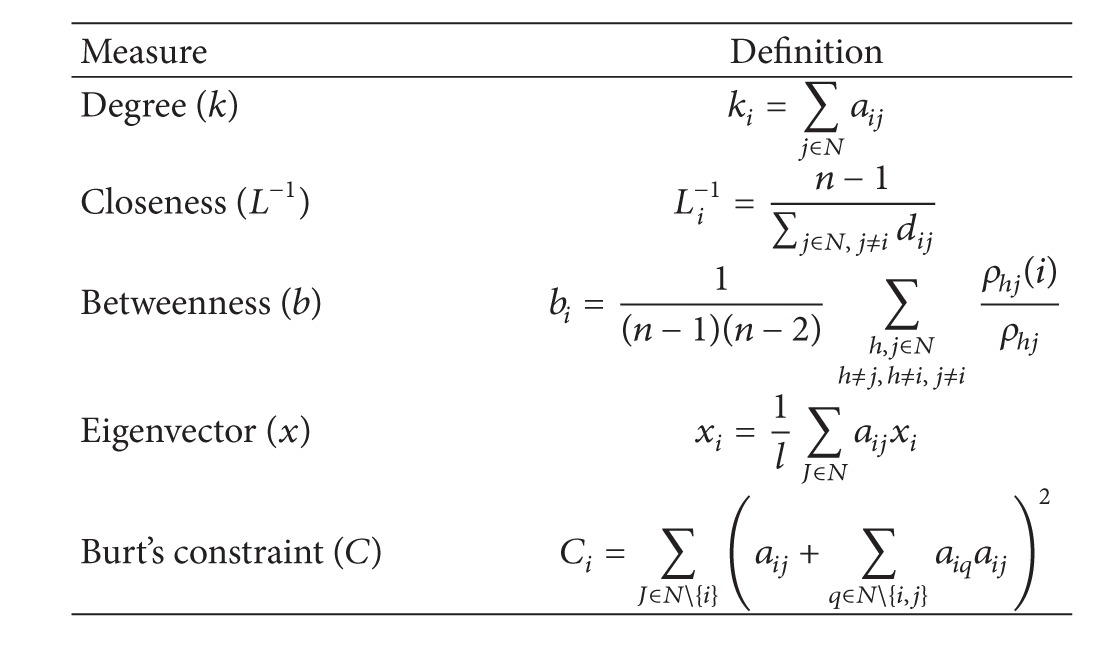

**Table 2:** Typical developing versus ADHD classification: sensitivity, specificity, and score for each centrality descriptor.

Typical Developing versus ADHD																				
	Descriptor	Cut-off	Peking			Kennedy Krieger			NeuroIMAGE			New York			OHSU			All sites		
			Spec	Sens	Score	Spec	Sens	Score	Spec	Sens	Score	Spec	Sens	Score	Spec	Sens	Score	Spec	Sens	Score
Unweighted graph	Closeness	0.1	100%	0%	50%	100%	0%	50%	100%	0%	50%	100%	0%	50%	100%	0%	50%	100%	0%	50%
		0.15	100%	0%	50%	100%	0%	50%	100%	0%	50%	100%	0%	50%	100%	0%	50%	100%	0%	50%
		0.25	100%	0%	50%	100%	0%	50%	100%	0%	50%	100%	0%	50%	100%	0%	50%	100%	0%	50%
	Between	0.1	61%	37%	49%	80%	24%	52%	52%	47%	50%	59%	56%	58%	45%	37%	41%	62%	45%	54%
		0.15	59%	40%	50%	72%	19%	46%	61%	47%	54%	59%	55%	57%	52%	40%	46%	63%	44%	54%
		0.25	63%	38%	51%	77%	19%	48%	**78%**	**47%**	**63%**	57%	60%	59%	48%	31%	40%	61%	44%	52%
	Degree	0.1	66%	44%	55%	82%	19%	51%	43%	42%	43%	59%	59%	59%	50%	43%	46%	63%	43%	53%
		0.15	65%	50%	57%	82%	24%	53%	57%	47%	52%	**60%**	**63%**	**62%**	50%	37%	44%	63%	40%	51%
		0.25	61%	49%	55%	77%	19%	48%	**61%**	**63%**	**62%**	**58%**	**68%**	**63%**	48%	31%	40%	65%	44%	54%

Weighted graph	Closeness	—	62%	35%	48%	87%	5%	46%	57%	42%	49%	46%	51%	48%	62%	54%	58%	65%	31%	48%
	Between	—	62%	44%	53%	85%	0%	43%	48%	37%	42%	52%	58%	55%	**83%**	**63%**	**73%**	62%	43%	53%
	Degree	—	59%	44%	51%	80%	29%	54%	**61%**	**68%**	**65%**	**60%**	**66%**	**63%**	55%	37%	46%	62%	50%	56%
	EVC	—	59%	38%	49%	85%	19%	52%	61%	37%	49%	**58%**	**68%**	**63%**	57%	31%	44%	74%	41%	58%
	Burt	—	100%	0%	50%	100%	0%	50%	100%	0%	50%	100%	0%	50%	100%	0%	50%	100%	0%	50%

**Table 3:** ADHD types classification: sensitivity, specificity, and score for each centrality descriptor. Note: the accuracy measures could not be obtained at the NeuroIMAGE site due to the small number of ADHD-combined subjects.

Inattentive ADHD versus Combined ADHD																				
	Descriptor	Cut-off	Peking			Kennedy Krieger			NeuroIMAGE			New York			OHSU			All sites		
			Spec	Sens	Score	Spec	Sens	Score	Spec	Sens	Score	Spec	Sens	Score	Spec	Sens	Score	Spec	Sens	Score
Unweighted graph	Closeness	0.1	100%	0%	50%	100%	0%	50%	—	—	—	100%	0%	50%	100%	0%	50%	0%	100%	50%
		0.15	100%	0%	50%	100%	0%	50%	—	—	—	100%	0%	50%	100%	0%	50%	100%	0%	50%
		0.25	100%	0%	50%	100%	0%	50%	—	—	—	100%	0%	50%	100%	0%	50%	100%	0%	50%
	Between	0.1	34%	69%	52%	**94%**	**40%**	**67%**	—	—	—	63%	37%	50%	**96%**	**42%**	**69%**	62%	45%	54%
		0.15	31%	71%	51%	**94%**	**40%**	**67%**	—	—	—	58%	33%	45%	**96%**	**50%**	**73%**	58%	47%	53%
		0.25	41%	69%	55%	88%	0%	44%	—	—	—	63%	37%	50%	**91%**	**42%**	**66%**	64%	46%	55%
	Degree	0.1	45%	61%	53%	**94%**	**40%**	**67%**	—	—	—	63%	35%	49%	**87%**	**58%**	**73%**	60%	49%	54%
		0.15	28%	71%	50%	94%	20%	57%	—	—	—	66%	33%	49%	**78%**	**75%**	**77%**	60%	55%	58%
		0.25	34%	71%	53%	88%	20%	54%	—	—	—	64%	42%	53%	**74%**	**58%**	**66%**	**67%**	**55%**	**61%**

Weighted graph	Closeness	—	34%	71%	53%	**100%**	**40%**	**70%**	—	—	—	63%	35%	49%	74%	33%	54%	60%	37%	49%
	Between	—	28%	78%	53%	100%	0%	50%	—	—	—	66%	37%	51%	74%	17%	45%	58%	46%	52%
	Degree	—	31%	73%	52%	88%	20%	54%	—	—	—	64%	37%	51%	**78%**	**67%**	**72%**	59%	51%	55%
	EVC	—	34%	71%	53%	69%	0%	34%	—	—	—	66%	37%	51%	**87%**	**58%**	**73%**	65%	46%	56%
	Burt	—	100%	0%	50%	100%	0%	50%	—	—	—	100%	0%	50%	100%	0%	50%	100%	0%	50%

## References

[1] Polanczyk G, De Lima MS, Horta BL, Biederman J, Rohde LA (2007). The worldwide prevalence of ADHD: a systematic review and metaregression analysis. *American Journal of Psychiatry*.

[2] American Psychiatric Association (2013). *Diagnostic and Statistical Manual of Mental Disorders*.

[3] Cuthbert BN, Insel TR (2013). Toward the future of psychiatric diagnosis: the seven pillars of RDoC. *BMC Medicine*.

[4] Insel T, Cuthbert B, Garvey M (2010). Research Domain Criteria (RDoC): toward a new classification framework for research on mental disorders. *American Journal of Psychiatry*.

[5] Linden DEJ (2012). The challenges and promise of neuroimaging in psychiatry. *Neuron*.

[6] Bullmore E, Sporns O (2009). Complex brain networks: graph theoretical analysis of structural and functional systems. *Nature Reviews Neuroscience*.

[7] Rubinov M, Sporns O (2010). Complex network measures of brain connectivity: Uses and interpretations. *NeuroImage*.

[8] Damoiseaux JS, Rombouts SARB, Barkhof F (2006). Consistent resting-state networks across healthy subjects. *Proceedings of the National Academy of Sciences of the United States of America*.

[9] de La Fuente A, Xia S, Branch C, Li X (2013). A review of attention-deficit/hyperactivity disorder from the perspective of brain networks. *Frontiers in Human Neuroscience*.

[10] Greicius M (2008). Resting-state functional connectivity in neuropsychiatric disorders. *Current Opinion in Neurology*.

[11] Biswal B, Yetkin FZ, Haughton VM, Hyde JS (1995). Functional connectivity in the motor cortex of resting human brain using echo-planar MRI. *Magnetic Resonance in Medicine*.

[12] Buckner RL, Andrews-Hanna JR, Schacter DL (2008). The brain's default network: anatomy, function, and relevance to disease. *Annals of the New York Academy of Sciences*.

[13] Klöppel S, Abdulkadir A, Jack CR, Koutsouleris N, Mourão-Miranda J, Vemuri P (2012). Diagnostic neuroimaging across diseases. *NeuroImage*.

[14] Vapnik VN (1998). *The Statistical Learning Theory*.

[15] HD-Consortium (2012). The ADHD-200 consortium: a model to advance the translational potential of neuroimaging in clinical neuroscience. *Frontiers in Systems Neuroscience*.

[16] Craddock RC, James GA, Holtzheimer PE, Hu XP, Mayberg HS (2012). A whole brain fMRI atlas generated via spatially constrained spectral clustering. *Human Brain Mapping*.

[17] Power JD, Fair DA, Schlaggar BL, Petersen SE (2010). The development of Human Functional Brain Networks. *Neuron*.

[18] Freeman LC (1979). Centrality in social networks conceptual clarification. *Social Networks*.

[19] Freeman LC (1977). A set of measures of centrality based on betweenness. *Sociometry*.

[20] Burt RS (2004). Structural holes and good ideas. *The American Journal of Sociology*.

[21] Mourão-Miranda J, Bokde ALW, Born C, Hampel H, Stetter M (2005). Classifying brain states and determining the discriminating activation patterns: Support Vector Machine on functional MRI data. *NeuroImage*.

[22] Sato JR, Fujita A, Thomaz CE (2009). Evaluating SVM and MLDA in the extraction of discriminant regions for mental state prediction. *NeuroImage*.

[23] Cubillo A, Halari R, Ecker C, Giampietro V, Taylor E, Rubia K (2010). Reduced activation and inter-regional functional connectivity of fronto-striatal networks in adults with childhood Attention-Deficit Hyperactivity Disorder (ADHD) and persisting symptoms during tasks of motor inhibition and cognitive switching. *Journal of Psychiatric Research*.

[24] Rubia K, Cubillo A, Smith AB, Woolley J, Heyman I, Brammer MJ (2010). Disorder-specific dysfunction in right inferior prefrontal cortex during two inhibition tasks in boys with attention-deficit hyperactivity disorder compared to boys with obsessive-compulsive disorder. *Human Brain Mapping*.

[25] Shaw P (2012). Attention-deficit/hyperactivity disorder and the battle for control of attention. *Journal of the American Academy of Child and Adolescent Psychiatry*.

[26] Bush G (2010). Attention-deficit/hyperactivity disorder and attention networks. *Neuropsychopharmacology*.

[27] Bush G (2011). Cingulate, frontal, and parietal cortical dysfunction in attention-deficit/hyperactivity disorder. *Biological Psychiatry*.

[28] Li X, Sroubek A, Kelly MS (2012). Atypical pulvinar-cortical pathways during sustained attention performance in children with attention-deficit/hyperactivity disorder. *Journal of the American Academy of Child and Adolescent Psychiatry*.

[29] Castellanos FX, Proal E (2012). Large-scale brain systems in ADHD: beyond the prefrontal-striatal model. *Trends in Cognitive Sciences*.

